# Clinical characteristics, AR gene variants, and functional domains in 64 patients with androgen insensitivity syndrome

**DOI:** 10.1007/s40618-022-01894-4

**Published:** 2022-08-16

**Authors:** Q. Liu, X. Yin, P. Li

**Affiliations:** Department of Endocrinology, Shanghai Children’s Hospital, School of Medicine, Shanghai Jiao Tong University, Shanghai, 200062 People’s Republic of China

**Keywords:** Androgen insensitivity syndrome (AIS), Genotype–phenotype correlation, Androgen receptor, Variant, Functional domain

## Abstract

**Background:**

Androgen insensitivity syndrome (AIS) is caused by abnormal androgen receptor (AR) genes that show variable genotypes and phenotypes. However, the correlation between genotype and phenotype is unclear.

**Methods:**

We retrospectively evaluated 64 patients with AIS at Shanghai Children's Hospital from 2015 to 2022. We analysed the clinical data of the patients, including hormone levels, AR gene variants, and functional domains.

**Results:**

Variants occurred in the 3 major functional domains in 56 patients, including 23 patients with complete androgen insensitivity syndrome (CAIS) and 33 with partial androgen insensitivity syndrome (PAIS). The incidence of nonscrotal fusion (*P* = 0.019) and proximal urethral opening (*P* = 0.0002) in the ligand-binding domain (LBD) group was higher than that in the non-LBD group. The phallus length in the LBD group was significantly shorter than that in the non-LBD group (*P* = 0.009). The external masculinization score (EMS) in the LBD group was significantly lower than that in the non-LBD group (*P* = 0.013). The levels of inhibin-B (INHB; *P* = 0.0007), basal luteinizing hormone (LH; *P* = 0.033), LH peak (*P* = 0.002), and testosterone (T) after human chorionic gonadotropin (HCG) stimulation (*P* = 0.001) in the LBD group were higher than those in the non-LBD group. There were 53 variants in 64 patients, including 42 reported and 11 novel AR variants, including p.Met247Arg, p.Asp266Glyfs*39, p.Arg362Serfs*140, p.Ala385Val, p.Glu541Asp, p.Pro613Leu, p.Pro695Leu, p.Asn757Asp, c.1616 + 1dup, c.1886-1G > A and exon 5–7 deletion.

**Conclusions:**

The EMS of patients with AIS in the LBD group was significantly lower than that in the non-LBD group. The phallus length was shorter, and the incidences of proximal urethral opening and nonscrotal fusion were higher, suggesting that the phenotypes in the LBD group were more severe. The levels of INHB, basal LH, peak LH, and T after HCG stimulation in the LBD group were higher than those in the non-LBD group, suggesting that androgen resistance in the LBD group was more severe. We identified 53 variants in 64 patients: 42 reported and 11 novel AR variants. These findings provide new and deeper insight into AIS diagnosis and genetic assessment of AIS.

**Supplementary Information:**

The online version contains supplementary material available at 10.1007/s40618-022-01894-4.

## Background

Androgen insensitivity syndrome (AIS) is an X-linked genetic disease that is commonly caused by 46, XY disorders of sex development (46, XY DSD) [1]. Androgen receptor (AR) is encoded by an 8-exon gene on chromosome Xq11–12 and is a protein of 920 amino acids. The protein contains three major functional domains: (1) the large N-terminal domain (NTD), which initiates the transcription of target genes; (2) the DNA-binding domain (DBD), which interacts with DNA; and (3) the ligand-binding domain (LBD), which first promotes the interaction of the receptor with heat shock proteins in the cytoplasm [2]. According to the clinical manifestations, AIS is mainly divided into complete AIS (CAIS), partial AIS (PAIS), and mild AIS (MAIS). Although the specific location of the variant in the protein impacts the severity of the phenotype, there is no genotype–phenotype correlation even in patients with the same variant. We investigated 64 patients with AIS to explore the clinical characteristics, serum sex hormone levels, and characteristics of the functional domains and AR gene variants. We also evaluated the phenotypic severity using the external masculinization score (EMS) [3].

## Patients and methods

### Patients

Informed parental consent, patient consent, and approval from the hospital ethics committee were obtained before the initiation of the study. Sixty-four patients with AIS were studied at the Department of Endocrinology of Shanghai Children's Hospital from 2015 to 2022, including 57 prepubertal children, 6 pubertal patients, and 1 6-month-old mini-pubertal infant. The patients met the following AIS criteria: 46, XY karyotype, sex-determining region Y (SRY)-positive (+), normal adrenal function, and AR variants. We investigated the clinical manifestations, family history, sex hormone levels, and AR gene sequences of the patients. In our study, AIS was classified as either complete androgen insensitivity syndrome (CAIS) or partial androgen insensitivity syndrome (PAIS) according to the clinical manifestations, and all patients with CAIS presented a female phenotype. According to the functional domain of the variant, it was mainly divided into either the LBD or non-LBD (i.e., DBD and NTD) groups. Thirty-seven patients with LBD variants (33 prepubertal children) and 19 with NTD and DBD variants (18 prepubertal children) were included in the study.

### External genital phenotype evaluation

We evaluated external genital phenotypes according to the external masculinization scores. External genitalia were scored separately for scrotal fusion, phallus size, location of the urethral meatus, and site of gonads. The EMS is the sum of the four subscores and can range from 0 to 12 as described by Ahmed SF et al. [3]. Phallus lengths were compared to the data from healthy Chinese children. Microphallus refers to a phallus body length less than − 2.5 standard deviations (SD) from the average length of the same age or sexual development status when the phallus is extended [4]. The phallus was placed in an extended state, and the length from the pubic symphysis to the top of the glans along the dorsal side of the phallus, excluding the foreskin length, was recorded as the phallus length. Cryptorchidism was confirmed by physical and ultrasound examinations and was classified as intra-abdominal or inguinal cryptorchidism based on the location of the testis [5]. Hypospadias was divided into proximal (severe), middle, and distal hypospadias according to the urethral opening position.

### Hormonal analysis

To evaluate testicular function, all patients underwent gonadotropin-releasing hormone (GnRH) stimulation to assess hypothalamic–pituitary–gonadal axis function and human chorionic gonadotropin (HCG) stimulation. GnRH stimulation was performed after intravenous injection of gonadorelin (Anhui Fengyuan Pharmaceutical Co., Ltd.) at 2.5 µg/kg (maximum dose 100 µg), and the serum luteinizing hormone (LH) and follicle-stimulating hormone (FSH) levels were measured 0, 30, 60 and 90 min after injection. The protocol for HCG stimulation included intramuscular injection of 1500 U HCG on 3 consecutive days with blood samples collected before the first dose of HCG on Days 1, 2 and 3 and then on Day 4. Sex hormones included anti-Müllerian hormone (AMH), inhibin-B (INHB), sex hormone-binding globulin (SHBG), Oestradiol (E2), basal testosterone (T), basal dihydrotestosterone (DHT), basal LH, peak LH, basal FSH, peak FSH, DHT (and T) after human chorionic gonadotropin (HCG) stimulation. Serum LH and FSH concentrations were evaluated using LH and FSH detection kits (Beckman Coulter) and measured with an automatic immunoluminescence analyser (UnicelDxI 800). Serum AMH and INHB levels were detected using solid-phase sandwich enzyme-linked immunosorbent assays (ELISAs) purchased from Guangzhou Kangrun Biotechnology Co., Ltd. T and DHT levels were evaluated by ELISA and measured using a Polar ELx800 microplate reader (USA). The minimum and maximum detection limits of AMH were 0.01 ng/mL and 25 ng/mL, respectively. In addition, if AMH exceeded the measurement range, it would be diluted for detection. The minimum and maximum detection limits of INHB were 10 pg/mL and 1300 pg/mL, respectively. The minimum and maximum detection limits of T were 0.35 nmol/L and 55.5 nmol/L, respectively. The minimum and the maximum detection limits of DHT were 1.55 pg/mL and 2500 pg/mL, respectively. The minimum and the maximum detection limits of LH were 0.2 IU/L and 250 IU/L, respectively. The minimum and the maximum detection limits of FSH were 0.2 IU/L and 200 IU/L, respectively. The minimum and the maximum detection limits of E2 were 55.07 pmol/L and 19,089 pmol/L, respectively.

### AR gene analysis

Exons 1–8 of AR were amplified, and next-generation sequencing was used to screen for variants. We performed searches within the Human Gene Mutation Database (http://www.hgmd.cf.ac.uk), the androgen mutation database (http://androgendb.mcgill.ca), and ClinVar (https://www.ncbi.nlm.nih.gov/clinvar/). SIFT and Mutation Taster software were used to perform pathogenicity analysis of the amino acid changes caused by the variants. The American College of Medical Genetics and Genomics (ACMG) criteria were used to classify variants, and I-TASSER (https://zhanggroup.org/I-TASSER/) was used to predict the protein structure of the AR variant. PyMOL software (https://pymol.org/2/) was used to analyse protein structural changes.

### Statistical analysis

SPSS 26.0 software (International Business Machines Corporation) was used to analyse the data. Nonparametric data were analysed with the Mann–Whitney *U* test and are presented as median values (25th and 75th percentiles). Fisher’s exact test was used to compare the incidence of clinical manifestations. Statistical significance was set at *P* < 0.05.

## Results

### Clinical manifestations

The cases in this study included 57 prepubertal children, 6 pubertal patients, and 1 6-month-old mini-pubertal infant. In addition to microphallus, the common manifestations in PAIS patients include hypospadias, cryptorchidism, and bifid scrotum. Although all PAIS patients had microphalli, only three patients had isolated microphalli. The incidence of the patient being raised as a female, CAIS, and proximal urethral opening (*P* = 0.0002) in the LBD group was higher than those in the NTD and DBD groups, whereas the incidence of cryptorchidism and scrotal fusion (*P* = 0.019) was lower (Table 1). The phallus length in the LBD group was significantly shorter than that in the NTD and DBD groups (0.3 vs. 1.1 cm; *P* = 0.009). EMS in the LBD group was significantly lower than that in the NTD and DBD groups (2 vs. 5.25; *P* = 0.013) (Table 2). Gynaecomastia occurred in three pubertal PAIS patients and breast development occurred in three CAIS patients with an EMS range of 1–6 (Table 3).

**Table 1:** Incidence of clinical manifestations in AIS patients

Clinical manifestation	LBD group	DBD and NTD group	Total	*P* value
Female	19 (51.35%)	6 (31.58%)	25	0.256
Male	18 (48.65%)	13 (68.42%)	31	
CAIS	17 (45.95%)	6 (31.58%)	23	0.394
PAIS	20 (54.05%)	13 (68.42%)	33	
Nonscrotal fusion	28 (75.68%)	8 (42.11%)	36	0.019
Scrotal fusion	9 (24.32%)	11 (57.89%)	20	
Cryptorchidism	24 (64.86%)	13 (68.42%)	37	> 0.999
Noncryptorchidism	13 (35.14%)	6 (31.58%)	19	
Proximal urethral opening	37 (100%)	12 (63.16%)	49	0.0002
Nonproximal urethral opening	0 (0)	7 (36.84%)	7

**Table 2:** Clinical manifestations and hormone levels in prepubertal patients with AIS

Hormones	LBD group (*n* = 33)	DBD and NTD groups (*n* = 18)	*P* Value
Age (year)	1.8 (1.1–3.3)	2.6 (1.5–7.4)	0.074
Penis length (cm)	0.3 (0–0.8)	1.1 (0.3–1.85)	0.009
EMS	2 (2–3)	5.25 (2–7.25)	0.013
AMH (ng/mL)	152.2 (27.43–335.7)	78.57 (22.28–152.8)	0.168
INHB (pg/mL)	319.5 (207.4–373.3)	138.1 (100.4–229.4)	0.0007
Basal LH (IU/L)	0.9 (0.55–1.73)	0.41 (0.24–1.31)	0.033
Peak LH (IU/L)	23.76 (13.54–30.46)	9.67(4.15–19.03)	0.002
Basal FSH (IU/L)	1.81 (1.47–3.39)	3.96 (1.11–5.12)	0.509
Peak FSH (IU/L)	6.28 (3.36–13.78)	10.7 (4.61–17.32)	0.174
Basal T (nmol/L)	0.88 (0.35–2.05)	0.35 (0.35–1.05)	0.095
T after HCG stimulation (nmol/L)	16 (9.72–21.85)	8.87 (5.33–9.93)	0.001
Basal DHT (pg/ml)	81.52 (40.97–125.4)	64.34 (28.26–172.6)	0.964
DHT after HCG stimulation (pg/ml)	237.3 (169.9–290.4)	183.3 (80.57–376.1)	0.330
T:DHT ratio	13.38 (6.44–18.13)	6.59 (4.58–12.31)	0.055
SHBG (nmol/l)	129.2 (105.4–157.9)	113.3 (86.55–162.5)	0.466

**Table 3:** Clinical manifestations and hormone levels in the mini-pubertal patient and six pubertal patients with AIS

Case	Age (year)	EMS	Breast development or gynaecomastia	AMH (ng/mL)	INHB (pg/mL)	LH (IU/L)	FSH (IU/L)	T(nmol/L)	E2(pmol/L)
C1	0.6	2	B1	25.53	636.36	0.89	2.15	2.95	< 55
C2	11.3	1.5	B4	57	231.12	19.5	15.2	15.8	73.4
C3	11.9	2	B3	47.86	385.41	3.56	5.74	4.8	76
C4	13.8	1	B5	23.87	494	24.86	2.28	32.8	141
P5	13.9	2	B3	21.4	266.56	11.88	7.38	43.47	79
P6	12.7	6	B3	24.98	213.25	2.83	2.52	7	73
P7	12.5	6	B3	6.22	129.71	2.78	4.85	8.25	65.6

### Hormones

We compared the hormone levels of 51 prepubescent patients (33 in the LBD group and 18 in the NTD and DBD groups). There was no significant difference in AMH, SHBG, basal FSH, basal T, basal DHT, peak FSH, or DHT hormone levels between the two groups after HCG stimulation. The levels of INHB (*P* = 0.0007), basal LH (*P* = 0.033), peak LH (*P* = 0.002), and T after HCG stimulation (*P* = 0.001) in the LBD group were higher than those in the NTD and DBD groups (Table 2). The levels of LH and testosterone in two patients (C4 and P5) were above the normal range. Additionally, the LH level of one patient (C2) was above the normal range, whereas the LH and testosterone levels of other patients were in the normal range (Table 3).

### Molecular studies

There were 30 LBD variants (including 29 missense variants and 1 frameshift variant) in 37 patients, 11 NTD variants (including 5 missense, 2 nonsense, and 3 frameshift variants, and 1 duplication variant) in 13 patients, 6 missense DBD variants in 6 patients, 2 exon deletions in 3 patients, and 4 intron variants in 5 patients. There were 53 variants (40 missense, 2 nonsense, 4 frameshift, 4 intron, and 2 exon deletion variants) in 64 patients, including 42 previously reported and 11 novel AR variants (Table 4). Patients with the p.Gln60*, p.Arg841His, and p.Arg856His variants developed CAIS and PAIS. Although two PAIS patients with the c.2450–42G > A variant exhibited a female phenotype, one patient had cryptorchidism, whereas the other had testicles in the scrotum.

**Table 4:** Novel AR gene variants in patients with AIS

NO	AIS	EMS	Case	Exon	Domain	Variant	Amino acid changes	Type	Pathogenicity	SIFT	MutationTaster
1	P	9	1	1	NTD	c.740 T > G	p.Met247Arg	Missense	Vus	0.0	0.996
2	C	1	1	1	NTD	c.796dup	p.Asp266Glyfs*39	Frameshift	P	U	U
3	C	2	1	1	NTD	c.1082_1083insC	p.Arg362Serfs*140	Frameshift	P	U	U
4	P	9	1	1	NTD	c.1154C > T	p.Ala385Val	Missense	Vus	0.002	0.570
5	P	6	1	2	DBD	c.1623G > T	p.Glu541Asp	Missense	Vus	0.494	0.980
6	P	4	1	3	DBD	c.1838C > T	p.Pro613Leu	Missense	Vus	0.003	1
7	P	3	1	4	LBD	c.2084C > T	p.Pro695Leu	Missense	LP	0.007	1
8	P	2	1	5	LBD	c.2269A > G	p.Asn757Asp	Missense	LP	0.014	1
9	C	2	1	N	Intron	c.1616 + 1dup	U	Intron	LP	U	U
10	C	2	1	N	Intron	c.1886-1G > A	U	Intron	P	U	U
11	P	1.5	1	5–7	Other	Exon 5–7 del	U	Deletion	P	U	U

Case, number of patients with the variant; LBD, ligand-binding domain; NTD, N-terminal domain; DBD, DNA-binding domain; C, CAIS; P, PAIS; LP, likely pathogenic; P, pathogenic; U, unknown.

**Fig. 1:**
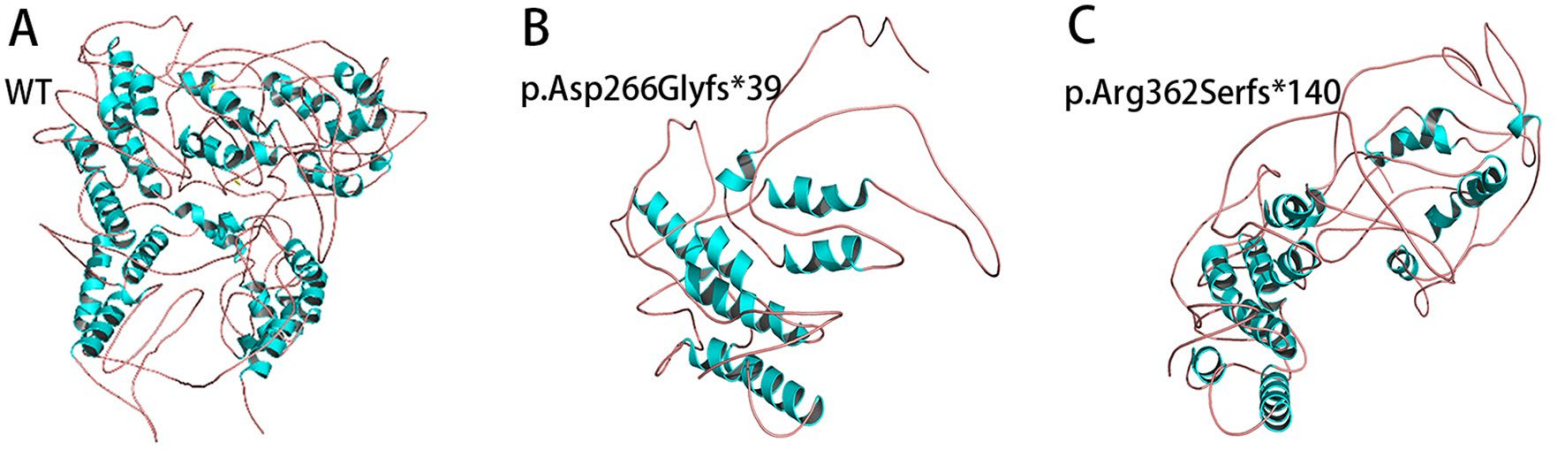
Two novel frameshift variants lead to changes in protein spatial structure

**Fig. 2:**
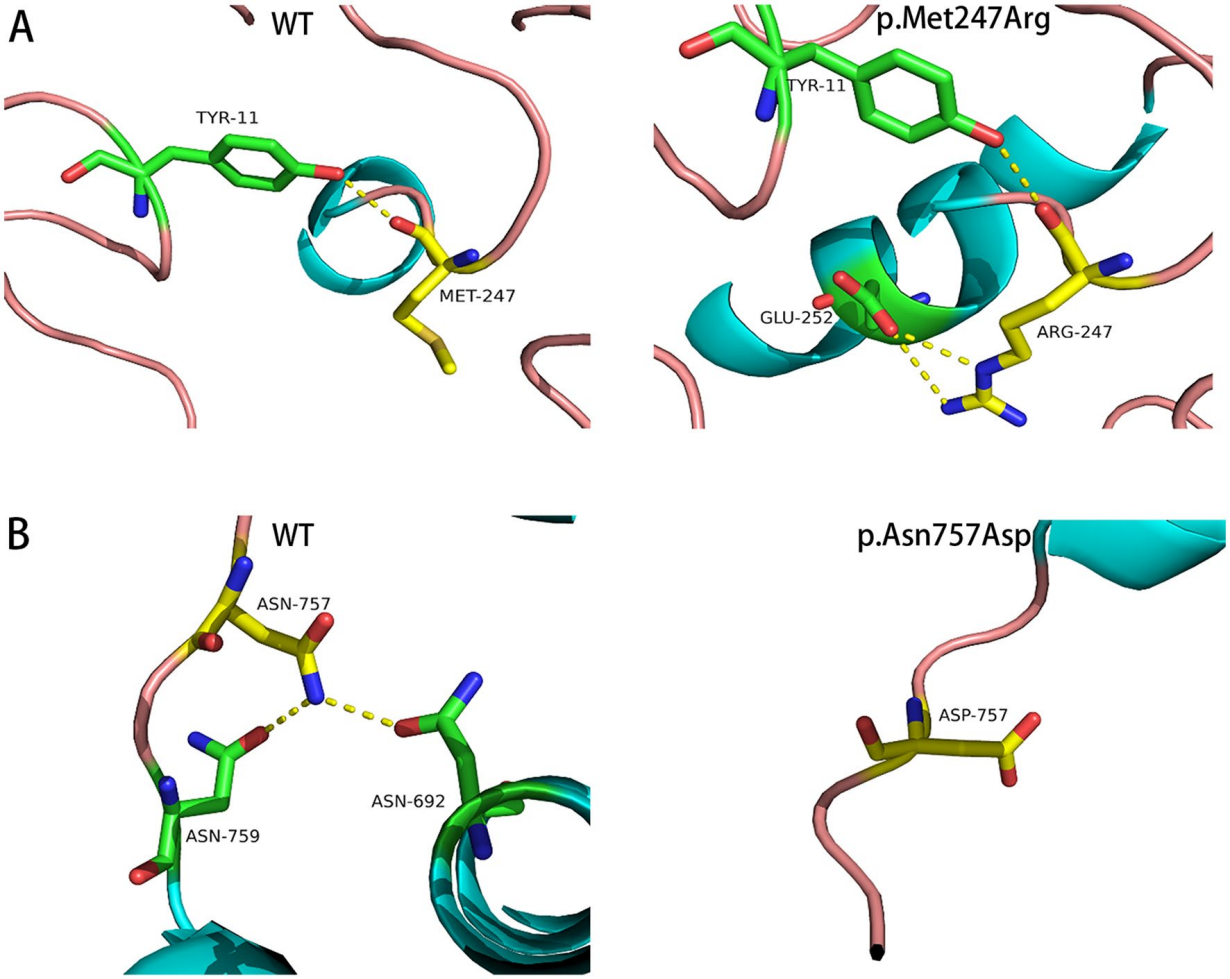
Two novel missense variants lead to changes in spatial protein structure and affect the interaction between amino acids

Compared with the WT protein spatial structure, the p.Asp266Glyfs*39 variant resulted in the loss of 19 α-helices (Fig. 1B), and the p.Arg362Serfs*140 variant resulted in the loss of 12 α-helices (Fig. 1C). The p.Ala385Val, p.Glu541Asp, p.Pro613Leu, and p.Pro695Leu variants did not affect the interaction between amino acids. In contrast, the p.Met247Arg and p.Asn757Asp variants affected the interaction between amino acids and protein function. The p.Met247Arg variant led to substitution of the nonpolar uncharged methionine residue with a large, polar, and positively charged arginine residue at position 247. This residue could form a hydrogen bond with the negatively charged glutamic acid residue at position 252 (Fig. 2A). The p.Asn757Asp variant led to the replacement of polar and uncharged asparagine amino residues with small, polar, and negatively charged asparagine at position 757. This residue could not form hydrogen bonds with the uncharged and polar asparagine residue at position 759 or the uncharged and polar asparagine residue at position 692 (Fig. 2B).

## Discussion

The EMS of AIS patients in the LBD group was significantly lower than that in the DBD and NTD groups, and the phallus length was also shorter. Furthermore, the incidences of proximal urethral opening and nonscrotal fusion were higher, suggesting a relatively severe phenotype in the LBD group. The basal and peak LH levels were 2.2 and 2.5 times that of the non-LBD group, respectively. The T after HCG stimulation was 1.8 times that of the non-LBD group. The levels of basal LH, peak LH, and T after HCG stimulation in the LBD group were higher than those in the non-LBD group, suggesting that androgen resistance in the LBD group was more severe. FSH levels are usually normal, which might be attributed to the regulation of gonadal INHB [6]. The level of SHBG in patients with AIS was within the normal range, which is consistent with findings in previous studies [7]. AMH and INHB serve as biological markers of testes development in patients with DSD [8]. The INHB in the LBD group was higher, which might be due to higher gonadotropin and T after HCG stimulation in the LBD group. A previous study showed that a T: DHT ratio above 10 was helpful in the diagnosis of 5α-reductase 2 deficiency [9], but the proportion of T: DHT ratios above 10 (29/57) was not low in this study. Therefore, the T: DHT ratio could not be used alone to distinguish AIS from 5α-reductase 2 deficiency.

The missense variant was most common in our study, and the variant rate of exon 1 was the highest among the eight exons. The p.Leu57dup, p.Gln58Leu and p.Asp266Asn variants are reportedly pathogenic in the NTD [10–12]. The novel p.Asp266Glyfs*39 variant resulted in the loss of 19 α-helices, and the novel p.Arg362Serfs*140 variant resulted in the loss of 12 α-helices, disrupting the spatial structure of the protein. The two novel nonsense variants in our study all occurred in the NTD, and most frameshift variants (3/4) occurred in the NTD. Variants involving NTD usually lead to frameshift changes, which is consistent with previous reports [13]. In addition, the p.Met247Arg and p.Ala385Val variants are novel variants of NTD. Crystal structure evidence suggested that the p.Met247Arg variant could form a hydrogen bond with the negatively charged glutamic acid residue at position 252, affecting the interaction between the amino acids. Variants in the DBD region lead to impaired AR activation and changes in DNA-binding and/or dimerization activity [14]. The p.Arg608Gln variant is reportedly pathogenic in the DBD [15, 16]. Furthermore, the p.Glu541Asp and p.Pro613Leu variants in the DBD were novel in our study.

In this study, all mutations in the LBD except for one frameshift variant were missense variants. LBD encoded by exons 4–8 has specific ligand-binding sites for androgens, various transcriptional coactivators, and the activation function 2 (AF-2) region [17, 18]. Missense variants in the LBD can disrupt AR protein function and lead to CAIS [19]. The patients in the LBD group had lower EMS and higher hormone levels, indicating that the variant in the LBD damaged AR function more seriously than those in other domains. The p.Arg753Gln variant is reportedly pathogenic [20, 21]. Exon 4 is the first exon of LBD, and the p.Pro695Leu variant in this exon is novel. The p.Asn757Asp variant is a novel variant of exon 5 and is located in the nuclear hormone receptor-binding region of the AR protein. The crystal structure evidence showed that it could not form hydrogen bonds with the uncharged and polar asparagine residues at position 759 and 692, thereby changing the protein structure.

In addition to the variant of the three functional domains, we identified a deletion variant of exon 2. The base contained in exon 2 is an integral multiple of non-3, and its deletion may lead to changes in the reading frame [22]. The deletion variant of exons 5–7 of the AR gene is novel and has not been recorded in the unaffected people database. However, HGMD has been recorded in many reports of large fragment deletions and disease [23, 24]. The intron variant c.1616 + 1dup was a repeated novel variant of one base pair, which changed the highly conserved donor splicing site of transcript exon 1 and introduced a new donor splicing site at 1 bp after the original donor splicing site, resulting in abnormal splicing and a change in the function of the protein product encoded by the AR gene. The intron c.1886-1G > A variant was novel and changed the highly conserved receptor splicing site of exon 4 of the transcript, resulting in abnormal splicing and a change in the function of the protein product encoded by the AR gene. Another splicing variant, c.1886-1G > T, at the same base position has been reported in several patients with AIS [22, 25]. c.1768 + 1G > C and c.2450-42G > A variants are reportedly pathogenic [26, 27]. The two sisters with PAIS with the c.2450-42G > A variant exhibited different phenotypes, suggesting that the phenotypes of AIS patients with the same gene variant may be different, even in the same family. Therefore, the genotype–phenotype correlation between patients has not been precisely established.

## Conclusions

In this study, we demonstrated that the EMS of patients with AIS in the LBD group was significantly lower than that in the DBD and NTD groups. The phallus length was shorter, and the incidence of proximal urethral opening and nonscrotal fusion was higher. INHB, basal LH, LH peak, and T levels after HCG stimulation in the LBD group were higher than those in the NTD and DBD groups. We identified 42 reported and 11 novel AR variants. These findings provide deeper insight into the clinical and genetic assessment of AIS.

## Supplementary Information

Below is the link to the electronic supplementary material.

Supplementary file1 (DOCX 25 KB)

## Data Availability

All data generated or analysed during this study are included in this published article. The datasets used and/or analysed during the current study are available from the corresponding author upon reasonable request.

## Abbreviations

AR: Androgen receptor
AIS: Androgen insensitivity syndrome
CAIS: Complete androgen insensitivity syndrome
PAIS: Partial androgen insensitivity syndrome
DSD: Disorders of sex development
EMS: External masculinization score
SRY: Sex-determining region Y
ACMG: American College of Medical Genetics and Genomics
INHB: Inhibin B
SHBG: Sex hormone-binding globulin
LH: Luteinizing hormone
FSH: Follicle-stimulating hormone
T: Testosterone
DHT: Dihydrotestosterone
E2: Oestradiol
HCG: Human chorionic gonadotropin
AMH: Anti-Mullerian hormone
LBD: Ligand-binding domain
NTD: N-terminal domain
DBD: DNA-binding domain
AF: Activation function
