# Supplementary material for: Clinical characteristics, AR gene variants, and functional domains in 64 patients with androgen insensitivity syndrome
Source: J Endocrinol Invest. 2022 Aug 16;46(1):151–8. doi: 10.1007/s40618-022-01894-4 (PMC9829593; doi:10.1007/s40618-022-01894-4)
Supplement: Supplementary file 1 — Supplementary file1 (DOCX 25 KB) [file 40618_2022_1894_MOESM1_ESM.docx]

**Supplemental material**

**Table 5.** Reported AR gene variants in patients with AIS.

| **NO.** | **AIS** | **EMS** | **Case** | **Exon** | **Domain** | **Variant** | **Amino acid changes** | **Type** | **Pathogenicity** | **SIFT** | **MutationTaster** |
| --- | --- | --- | --- | --- | --- | --- | --- | --- | --- | --- | --- |
| 1 | P | 7 | 1 | 1 | NTD | c.170_172dup | p.Leu57dup | Duplication | P | U | U |
| 2 | 2P | 7.5 | 2 | 1 | NTD | c.170T>A | p.Leu57Gln | Missense | Vus | 1.0 | 1 |
| 3 | P | 5.5 | 1 | 1 | NTD | c.173A>T | p.Gln58Leu | Missense | vus | 0.01 | 1 |
| 4 | 1C,1P | 5 | 2 | 1 | NTD | c.178C>T | p.Gln60* | Nonsense | P | U | 1 |
| 5 | P | 7 | 1 | 1 | NTD | c.796G>A | p.Asp266Asn | Missense | vus | 0.08 | 0.982 |
| 6 | P | 2 | 1 | 1 | NTD | c.1063G>T | p.Glu355* | Nonsense | P | U | 1 |
| 7 | C | 2 | 1 | 1 | NTD | c.1415_1416insCGGC | p.Gly472fs | Frameshift | P | U | U |
| 8 | C | 1 | 1 | 2 | DBD | c.1684A>T | p.Ile562Phe | Missense | LP | 0.001 | 1 |
| 9 | P | 4 | 1 | 2 | DBD | c.1705G>T | p.Gly569Trp | Missense | LP | 0.0 | 1 |
| 10 | P | 5 | 1 | 3 | DBD | c.1823G>A | p.Arg608Gln | Missense | P | 0.001 | 1 |
| 11 | C | 2 | 1 | 3 | DBD | c.1858T>C | p.Cys620Arg | Missense | LP | 0.0 | 1 |
| 12 | C | 2 | 1 | 4 | LBD | c.2024T>A | p.Leu675Gln | Missense | LP | 0.0 | 1 |
| 13 | C | 2 | 1 | 4 | LBD | c.2086G>A | p.Asp696Asn | Missense | P | 0.0 | 1 |
| 14 | P | 6 | 1 | 4 | LBD | c.2104C>A | p.Leu702Ile | Missense | LP | 0.002 | 1 |
| 15 | 2C | 1.5 | 2 | 4 | LBD | c.2107T>C | p.Ser703Pro | Missense | LP | 0.001 | 0.998 |
| 16 | C | 1 | 1 | 4 | LBD | c.2117A>G | p.Asn706Ser | Missense | P | 0.0 | 1 |
| 17 | P | 6 | 1 | 4 | LBD | c.2128G>A | p.Glu710Lys | Missense | p | 0.051 | 1 |
| 18 | P | 3 | 1 | 4 | LBD | c.2158G>A | p.Ala720Thr | Missense | Vus | 0.001 | 1 |
| 19 | P | 2 | 1 | 5 | LBD | c.2221T>A | p.Ser741Thr | Missense | LP | 0.037 | 1 |
| 20 | C | 1 | 1 | 5 | LBD | c.2258G>A | p.Arg753Gln | Missense | p | 0.001 | 1 |
| 21 | C | 2 | 1 | 5 | LBD | c.2290T>C | p.Tyr764His | Missense | LP | 0.006 | 1 |
| 22 | P | 3 | 1 | 5 | LBD | c.2296G>T | p.Ala766Ser | Missense | P | 0.0 | 1 |
| 23 | C | 2 | 1 | 6 | LBD | c.2324G>A | p.Arg775His | Missense | P | 0.0 | 1 |
| 24 | C | 2 | 1 | 6 | LBD | c.2338C>T | p.Arg780Trp | Missense | P | 0.149 | 1 |
| 25 | P | 3 | 1 | 6 | LBD | c.2343G>A | p.Met781Ile | Missense | P | 0.088 | 1 |
| 26 | C | 2 | 1 | 6 | LBD | c.2351A>G | p.Gln784Arg | Missense | LP | 0.85 | 1 |
| 27 | P | 5 | 1 | 6 | LBD | c.2441T>A | p.Phe814Tyr | Missense | LP | 0.001 | 1 |
| 28 | P | 3 | 1 | 7 | LBD | c.2521C>T | p.Arg841Cys | Missense | P | 0.0 | 1 |
| 29 | 2C,1P | 2.3 | 3 | 7 | LBD | c.2522G>A | p.Arg841His | Missense | P | 0.015 | 1 |
| 30 | 2P | 6 | 2 | 7 | LBD | c.2531C>A | p.Ala844Glu | Missense | LP | 0.209 | 1 |
| 31 | C | 2 | 1 | 7 | LBD | c.2546dup | p.Asn849Lysfs*32 | Frameshift | p | U | U |
| 32 | 1C,1P | 3.5 | 2 | 7 | LBD | c.2567G>A | p.Arg856His | Missense | P | 0.005 | 1 |
| 33 | 3P | 4.5 | 3 | 8 | LBD | c.2612C>G | p.Ala871Gly | Missense | P | 0.01 | 1 |
| 34 | P | 6 | 1 | 8 | LBD | c.2612C>T | p.Ala871Val | Missense | P | 1.0 | 1 |
| 35 | P | 3 | 1 | 8 | LBD | c.2638G>T | p.Asp880Tyr | Missense | LP | 0.812 | 1 |
| 36 | C | 2 | 1 | 8 | LBD | c.2645T>C | p.Leu882Pro | Missense | P | 0.0 | 1 |
| 37 | C | 1 | 1 | 8 | LBD | c.2678C>T | p.Pro893Leu | Missense | P | 0.0 | 1 |
| 38 | C | 1 | 1 | 8 | LBD | c.2740C>G | p.Pro914Ala | Missense | LP | 0.077 | 1 |
| 39 | P | 2 | 1 | 8 | LBD | c.2740C>T | p.Pro914Ser | Missense | LP | 0.028 | 1 |
| 40 | C | 1 | 1 | N | Intron | c.1768+1G>C | U | Intron | P | U | U |
| 41 | 2P | 2.5 | 2 | N | Intron | c.2450-42G>A | U | Intron | P | U | U |
| 42 | 2C | 1.75 | 2 | 2 | Other | Exon 2 del | U | Deletion | P | U | U |

Case, number of patients with the variant; LBD, ligand-binding domain; NTD, N-terminal domain; DBD, DNA-binding domain; C, CAIS; P, PAIS; LP, likely pathogenic; P, pathogenic; U, unknown.
